# Frequency of Lost to Follow-Up and Associated Factors for Patients with Rheumatic Diseases

**DOI:** 10.1371/journal.pone.0150816

**Published:** 2016-03-07

**Authors:** Ya-Chih Tien, Ying-Ming Chiu, Mei-Ping Liu

**Affiliations:** 1 Allergy, Immunology and Rheumatology Division, Department of Internal Medicine, Changhua Christian Hospital, Changhua City, Taiwan; 2 Department of Nursing, College of Medicine & Nursing, HungKuang University, Taichung City, Taiwan; Renal Division, Peking University First Hospital, CHINA

## Abstract

**Objective:**

To determine the frequency of lost to follow-up (LTFU) in the setting of usual care for outpatients with rheumatic diseases including RA, SLE, AS, and Ps/PsA, to explore the associated demographic factors, and to investigate the reasons for being LTFU from the original medical care.

**Methods:**

Patients registered between May 2011 and January 2014 at the rheumatology outpatient department of a medical center were included. Those who did not attend their scheduled appointment were defined as LTFU. Univariate and multivariate logistic regression were used to analyze the factors for being LTFU.

**Results:**

A total of 781 patients were enrolled, including 406 patients with RA, 174 with SLE, 136 with AS, and 65 with Ps/PsA. The frequency of LTFU was 23.9%, 25.9%, 35.3%, and 35.4%, respectively. The frequency of LTFU was significantly different between the four rheumatic diseases (p = 0.028). In multivariate logistic regression analysis, an older age increased being LTFU in the patients with RA (OR 1.02; 95% CI 1.00–1.04; p = 0.033), but reduced being LTFU in those with Ps/PsA (OR 0.96; 95% CI 0.92–0.99; p = 0.021). Female patients with SLE and Ps/PsA were more likely to be LTFU, although this did not reach statistical significance (p = 0.056 and 0.071, respectively). The most common reason for being LTFU was moving to other district hospitals from the original medical center due to convenience for the patients with RA and SLE, and stopping medication due to minimal symptoms for the patients with AS and Ps/PsA.

**Conclusions:**

The frequency of LTFU in patients with rheumatic diseases is high. Associated demographic factors included older age in RA, female gender in SLE and Ps/PsA, and younger age in Ps/PsA, with various reasons for being LTFU. Recognizing these associated factors and reasons for being LTFU may help to improve the attendance of patients and the quality of medical care.

## Introduction

For the majority of rheumatic diseases such as rheumatoid arthritis (RA), systemic lupus erythematosus (SLE), ankylosing spondylitis (AS), and psoriasis/psoriatic arthritis (Ps/PsA), current therapeutic regimes have been shown to slow disease progression [1–4]. Thus regular outpatient follow-up with medication and continuous monitoring of disease activity are required for successful control of the disease, and to prevent advanced organ damage [5,6]. However, the failure of patients to maintain regular follow-up is a major problem for clinical physicians [7]. Patients who are lost to follow-up (LTFU) without returning for continuous care may contribute to a considerable worsening of the disease, leading to organ damage and an increase in health care expenditure in many chronic diseases [8,9], including rheumatic diseases [10–12]. In addition, high societal costs are attributed to LTFU not only due to increased healthcare spending but also due to loss in productivity related to impairment of work capacity or daily activities when diseases are left untreated among those LTFU [13]. Thus, LTFU has emerged as a key indicator of treatment effectiveness.

The frequency and factors associated with LTFU have seldom been studied in rheumatic diseases [14]. A few studies have assessed LTFU in SLE and RA, and only some of these studies have addressed the factors related to LTFU [15–19]. However, no studies have investigated the factors related to LTFU in AS or Ps/PsA, and no studies have compared LTFU between different rheumatic diseases.

The aims of this study were to evaluate the frequency of LTFU from the original outpatient department among patients with different rheumatic diseases (RA, SLE, AS, Ps/PsA) in the setting of usual care of a medical center, and to analyze the demographic factors associated with LTFU and to summarize the common reasons for LTFU from the original outpatient department.

## Materials and Methods

### Study population

The study population included patients from our rheumatology outpatient department in a medical center in central Taiwan. Patients with RA, SLE, AS, or PS/PsA who registered between May 2011 and January 2014 were enrolled into this study. Data on their disease course, drug compliance, co-morbidities, and outpatient visits were recorded for analysis.

### Patient recall and definition of follow-up status

In our rheumatology outpatient department, the frequency of outpatient visits to physicians is every 1 to 3 months. After each visit, the physician makes the next appointment for each patient. In order to remind the patients to return to the appointment on schedule, and record the reason if they did not return despite being reminded, a call center was established to monitor the outpatient visit status of these patients. Accordingly, the patients were classified into different groups. The patients who regularly returned for their scheduled appointments on the correct date were defined as the regular follow-up (RFU) group. For the patients who did not attend their scheduled appointment, a telephone call was made by the call center to remind them of their appointment. Those who returned for their appointment after this call were still classified as the RFU group. The patients who could not be contacted or did not return to the original outpatient department despite being reminded by telephone were defined as the potential LTFU group. The potential LTFU group was then classified into three subgroups: true LTFU, defined as those who could not be contacted after two more telephone calls (a total of three calls); those with documented reasons for not coming back to the original outpatient department; and those who had died. For the patients who died, the causes of mortality were recorded after contacting their family or from chart records [20]. Once the patient was classified into the LTFU group, the call center stopped continuous follow-up and reminders. Therefore, each patient was classified into only one of the groups throughout this study, and no patient was counted more than once in each group. Thus we calculated the total number of patients in this cohort as the denominator, and the number of LTFU patients as the numerator to evaluate the frequency of LTFU. To avoid errors due to changes in contact information, the patients were ask to update their contact information including telephone number every year when they came to the hospital.

### Categories of reasons for being LTFU from the original outpatient department of the medical center

The patients in the potential LTFU subgroup with documented reasons were asked the open question, “Why didn’t you return for your appointment?” In order to collect objective answers from the patients, all questions and answers were recorded by the call center staff, independent of the physicians. The documented answers were then sent to two physicians for interpretation. Categories for the reasons were then interpreted by the two physicians independently based on the WHO adherence theory [21], and then modified according to our clinical scenario. If there was a disagreement in the category between two physicians, a third physician was asked to interpret the category and a consensus was reached.

### Statistical analysis

We used proportions to present the distribution of the frequency of potential LTFU in the patients with the four rheumatic diseases. Chi-squared tests were used to examine the association between rheumatic diseases and LTFU. Univariate and multivariate logistic regression models were used for each of the demographic factors to study the influence on LTFU, and the results were presented as odds ratio (OR) with 95% confidence interval (CI). P values less than 0.05 were considered to be significant. For those with documented reasons for being LTFU from the original rheumatology outpatient department, we summarized the most common reasons for the four rheumatic diseases separately. Statistical analysis was performed using SAS® software, version 9.2 for Windows (SAS Institute Inc., Cary, NC, USA).

### Ethics Statement

The study was reviewed and approved by the Institutional Review Board of Changhua Christian Hospital (IRB number 140611). The study is based on secondary analysis of information from electronic databases of the call center. All data are de-identified; all personal identifiers are removed and physicians have no access to identify patient information. Patients were not asked to provide informed consent for the use of data as the approvals of the ethics board listed above.

## Results

The cohort consisted of 781 outpatients, of whom 98 (12.55%) were new patients, while the remaining patients had visited our rheumatology outpatient department before the initiation of the study. There were 406 with RA, 174 with SLE, 136 with AS, and 65 with Ps/PsA. Table 1 summarizes the demographic data of the four groups.

**Table 1:** Demographic profile of the cohort patients.

	RA	SLE	AS	Ps/PsA
Number of patients, n (%)	406 (52)	174 (22.3)	136 (17.4)	65 (8.3)
Age, years, (mean±SD)	58.3±13.9	39.7±14.1	38.4±13.4	51.9±15.4
Female gender, n (%)	317 (78.1)	155 (89.1)	25 (18.4)	32 (49.2)

RA: rheumatoid arthritis, SLE: systemic lupus erythematosus, AS: ankylosing spondylitis, Ps/PsA: psoriasis/psoriatic arthritis

### Differences in the frequency of potential LTFU among the four rheumatic disorders

**Fig 1:**
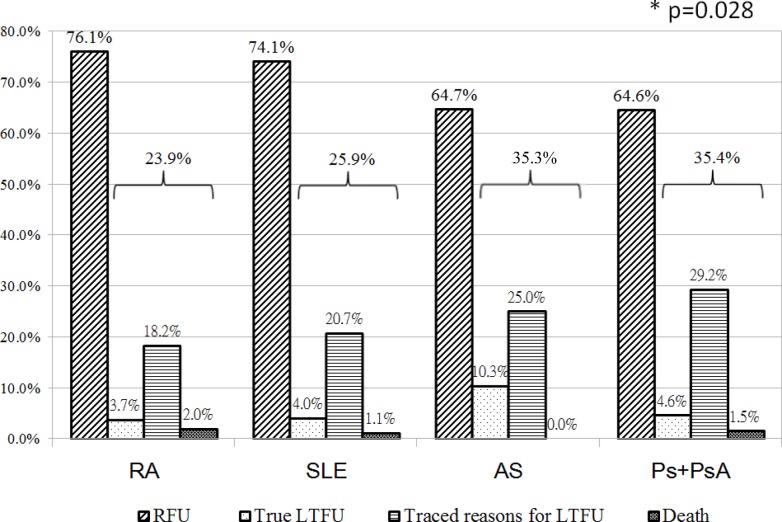
The frequency of LTFU in the four rheumatic diseases. *The p value (chi-squared test) evaluated the association between the four rheumatic diseases and being potential LTFU. The percentage (%) represent the proportion of follow-up status in each disease. The frequency of LTFU is represented by braces. RFU: regularly follow-up, LTFU: lost to follow-up.

Fig 1 illustrates the frequency of potential LTFU and the three subgroups of potential LTFU (true LTFU, traced reasons for LTFU, and death). The frequency of potential LTFU was higher in the patients with Ps/PsA (35.4%), followed by AS (35.3%), RA (23.9%) and SLE (23.9%). The frequency of potential LTFU was significantly different between the four rheumatic diseases (p = 0.028) (Fig 1). The frequency of true LTFU was also significantly different between the four rheumatic diseases (p = 0.0198).

### Associated factors with potential LTFU

Selected variables from the demographic data including age and gender were analyzed for their relationship with potential LTFU (Table 2). In multivariate logistic regression analysis, increasing age was associated with increased LTFU in the patients with RA (OR 1.02; 95% CI 1.00–1.04; p = 0.033), however increasing age was associated with a lower frequency of LTFU in the patients with Ps/PsA (OR 0.96; 95% CI 0.92–0.99; p = 0.021). Female patients with SLE and Ps/PsA were more likely to be LTFU, although this did not reach statistical significance (OR 7.38; 95% CI 0.95–57.29; p = 0.056 in SLE, OR 2.82; 95% CI 0.91–8.67; p = 0.071 in Ps/PsA).

**Table 2:** Baseline demographic characteristics associated with LTFU in the four rheumatic diseases.

		RFU	LTFU	Univariate		Multivariate	
				OR (95% CI)	P value ^a^	OR (95% CI)	P value ^a^
**RA**	No. of patients	309	97	—	—	—	**—**
	Age, years (mean±SD)	57.49±13.30	60.85±15.41	1.02 (1.00–1.04)	0.039*	1.02 (1.00–1.04)	0.033*
	Female, n (%)	239 (77.35)	78 (80.41)	1.20 (0.68–2.12)	0.525	1.27 (0.72–2.25)	0.415
**SLE**	No. of patients	129	45	—	—	—	**—**
	Age, years (mean±SD)	39.54±13.78	40.16±15.11	1.00 (0.98–1.03)	0.801	1.01 (0.98–1.03)	0.622
	Female, n (%)	111 (86.05)	44 (97.78)	7.13 (0.92–55.07)	0.060	7.38 (0.95–57.29)	0.056
**AS**	No. of patients	88	48	—	—	—	**—**
	Age, years (mean±SD)	38.98±13.41	37.44±13.55	0.99 (0.97–1.02)	0.522	0.99 (0.97–1.02)	0.548
	Female, n (%)	15 (17.05)	10 (20.83)	1.28 (0.53–3.12)	0.586	1.25 (0.51–3.07)	0.620
**Ps/PsA**	No. of patients	42	23	—	—	—	**—**
	Age, years (mean±SD)	54.88±16.30	46.52±12.40	0.96 (0.93–1.00)	0.042*	0.96 (0.92–0.99)	0.021*
	Female, n (%)	18 (42.86)	14 (60.87)	2.07 (0.74–5.85)	0.168	2.82 (0.91–8.67)	0.071

RA: rheumatoid arthritis, SLE: systemic lupus erythematosus, AS: ankylosing spondylitis, Ps/PsA: psoriasis/psoriatic arthritis, RFU: regularly followed-up, LTFU: lost to follow-up.

OR: odds ratio, CI: confidence interval.

^a^ P values were calculated using logistic regression analysis.

*A p value < 0.05 was considered to be significant.

### Traced reasons for LTFU from the original outpatient department of the medical center

**Fig 2:**
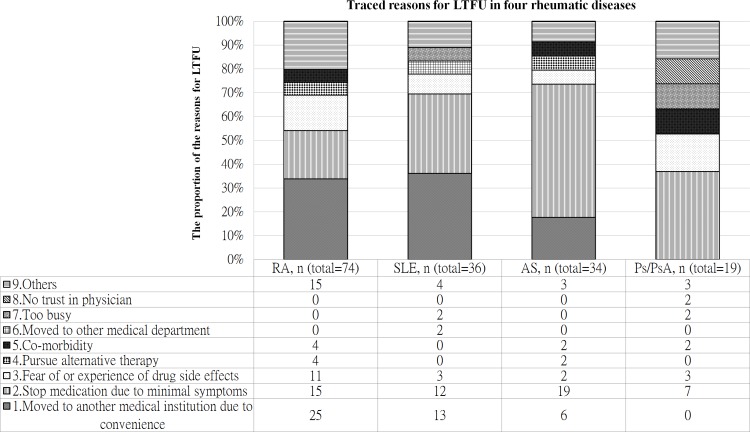
Traced reasons for LTFU. The figure illustrates the proportion of the reasons for LTFU in the four rheumatic diseases.

The main reasons for being LTFU from the original rheumatology outpatient department of our hospital for the patients with the four diseases are illustrated in Fig 2. Similar results were found among the RA and SLE patients, and the most common reason for being LTFU from the original outpatient department was moving to another medical institution due to convenience (from our medical center to district hospitals) (n = 25, 33.8% in RA; n = 13, 36.1% in SLE). The second most common reason was stopping medication due to minimal symptoms (n = 15, 20.3% in RA; n = 12, 33.3% in SLE), followed by a fear of or experience of drug side effects (n = 11, 14.9% in RA; n = 3, 8.3% in SLE). In the patients with AS, the most common reason for being LTFU from the original outpatient department was stopping medication due to minimal symptoms (n = 19, 55.9%), followed by moving to another medical institution due to convenience (n = 6, 17.3%). In the patients with Ps/PsA, the major reason for being LTFU from the original outpatient department was stopping medication due to minimal symptoms (n = 7, 36.8%), followed by a fear of or experience of drug side effects (n = 3, 15.8%).

## Discussion

To the best of our knowledge, this is the first study to compare the frequency of LTFU among different rheumatic diseases, including RA, SLE, AS, and Ps/PsA. By using clinical registry data, we present real world data from a medical center. We also identified differences in the frequency, associated demographic factors, and reasons for being LTFU from the original rheumatology outpatient department of a medical center in a clinical setting among patients with the four rheumatic diseases.

We found that the frequency of LTFU was higher in the patients with Ps/PsA (35.4%), and these LTFU patients were significantly younger than the RFU patients. Female patients with Ps/PsA also had a trend of being LTFU. It has been reported that female patients have less severe psoriasis [22]. We also found that the most common reason for being LTFU from the original outpatient department was due to stopping medications due to minimal symptoms. This may be because most younger, female patients have busy working schedules, and the minimal psoriasis symptoms meant they felt it was acceptable to ignore regular outpatient visits. Some studies have reported that treatment type is associated with patient adherence, suggesting that adherence is higher in patients receiving oral medication than topical therapy [23]. This may explain why there was a higher frequency of LTFU in the Ps/PsA group, as these patients have a higher rate of using topical rather than oral medication.

The patients with AS also had a higher frequency of LTFU (35.3%). Approximately half of the patients with AS (55.9%) did not attend their appointment due to minimal symptoms and an absence of daily dysfunction. This finding is similar to another single center study, in which the AS patients who were LTFU had a lower mean BASDAI score at baseline and a lower mean BASFI score at last visit. The reasons for non-attendance were good symptomatic status (72%), changing physicians (16.1%), and moving to another location (6.8%) [24]. This may suggest that the milder disease in AS patients tends to them being LTFU, and that physicians should pay more attention to these patients. Unlike the patients with Ps/PsA, there was no significant difference in age between the patients with AS in the RFU and LTFU groups. This may be because the average age of all of the patients with AS was young, and the age between the RFU and LTFU groups was similar.

In the patients with SLE, the frequency of LTFU in the current study was 25.9%, compared to 29% to 40% in previous studies [16,20]. These rates are difficult to compare because of differences in study design and the definition of LTFU [16,17,20]. In addition, the patients in the previous studies were younger [15,16], had more active disease [16,18], and more renal involvement [16], factors which have been found to be associated with LTFU in SLE. We found that female gender had a trend of being associated with an increased likelihood of being LTFU in the patients with SLE. It has been reported that female gender is associated with milder disease compared with male gender [25–29]. We also found that the most common reasons for being LTFU from the original outpatient department in our patients with SLE were moving to another medical institution due to convenience (from medical center to district hospitals) followed by stopping medication due to minimal symptoms. In Taiwan, the establishment of the National Health Insurance (NHI) has led to healthcare services being more widely available. Therefore, it is possible that the female patients with less severe disease in our study may have attended district hospitals, and thus were more likely to become LTFU from the original outpatient department of our medical center.

In the patients with RA, old age was found to be associated with a higher frequency of LTFU, although the age difference was only three years. The most common reasons for being LTFU from the original outpatient department were moving to another medical institution due to convenience, followed by minimal symptoms. Since the patients with RA were older, convenience may be an important consideration when choosing the hospital, and thus they were also more likely to become LTFU from the original outpatient department in our study.

According to policy in Taiwan, a medical center should be able to cater for a population of two million people, with a capacity of more than 500 beds and providing advanced medical care, education, and research. In contrast, a district hospital should be able to cater for a population of one hundred thousand people, with a capacity of 100 beds and the major goal of providing basic outpatient services and convenient medical care. Changhua Christian Hospital is a medical center in central Taiwan which offers over 60 clinical specialty and subspecialty departments, and provides comprehensive advanced medical care serving approximately 5,000 patient visits every day. Expectations of advanced medical services may prompt patients to visit our center, and some patients attend from a distance. However, LTFU may occur once those who attend from distance decide to return to their district hospital due to convenience after their disease has stabilized.

Another potentially interesting issue is the background of the NHI program in Taiwan which was established in 1995. This single-payer compulsory social insurance program now has a coverage rate as high as 99%, centralizes the disbursement of healthcare funds, and is administered by the government. The healthcare system has had a major impact on the population of Taiwan and has changed the behavior of seeking medical care in Taiwan. People can seek treatment with very low copayment, especially for those who qualify for a catastrophic illness card for chronic diseases such as RA and SLE, with the patients themselves just paying about 100 New Taiwan Dollars for each outpatient visit (around USD 3). Therefore, we believe that medical cost is not a major reason for LTFU in our study. In addition, the healthcare system is very accessible, and patients can freely choose to visit any doctor and hospital if they need medical care, either medical centers or district hospitals. Patients may also seek medical advice in a medical center initially, and then once the diagnosis has been confirmed and the disease stabilized, they tend to return to a nearby district hospital without informing or being referred by their original doctors, and thus become LTFU from the original medical center. Hence, this study not only evaluates the frequency of LTFU, but also reflects the needs of medical resources and the behavior of seeking medical care for patients based information from the NHI system in Taiwan. We think that to improve the attendance of patients and the quality of medical care, physicians should be alert to the biopsychosocial aspects of each patient, and tailor an exclusive model for each patient under the setting of different medical systems.

There are several limitations to this study. First, there is currently no standard definition of LTFU. We used our own definition to identify those who did not attend their scheduled appointment. However, without a standardized definition, it is difficult to evaluate whether differences between studies are real or an artifact due to different definitions. Second, although ethnicity has also been reported to be a factor for LTFU [15,16,17], this should not have affected our results due to the high homogeneity of ethnicity in Taiwan (> 98% of the population are Han Chinese). We were not able to examine other demographic variables such as employment, marital status, level of education, socioeconomic status, literacy, or religion, which have also been reported to be associated with non-adherence in previous studies. Third, the small number of patients limited our statistical analysis, thus some of the findings such as gender in the SLE and Ps/PsA patients only showed a small difference. Studies with a larger number of patients are warranted. Finally, we were unable to analyze the correlation between LTFU and diseased activity or severity in this study due to a lack of integral evaluation of disease activity in daily clinic practice. This limitation may diminish some of the potentially interesting findings about the frequency of LTFU.

In conclusion, the frequency of LTFU in outpatients with chronic rheumatic diseases was high. The factors associated with being LTFU included age and gender, with various reasons for being LTFU from the original outpatient department according to the disease. Early identification of the patients being LFTU may help to improve the healthcare and the attendance of the patients. However, these results may vary between different medical resources or insurance systems. How to assess, predict, and improve both follow-up rate and adherence at outpatient department is still a challenge for physicians.
